# *Fusobacterium nucleatum* facilitates proliferation and autophagy by activating miR-361-3p/NUDT1 axis through oxidative stress in hypopharyngeal squamous cell carcinoma

**DOI:** 10.1186/s12885-023-11439-4

**Published:** 2023-10-17

**Authors:** Hui-Ching Lau, Xiaohui Yuan, Huiying Huang, Ming Zhang, Chi-Yao Hsueh, Hongli Gong

**Affiliations:** 1 Department of Otorhinolaryngology, Eye & ENT Hospital, Fudan University, Shanghai, China; 2 Shanghai Key Clinical Disciplines of Otorhinolaryngology, Shanghai, PR China

**Keywords:** Hypopharyngeal squamous cell carcinoma, *Fusobacterium nucleatum*, miR-361-3p, Autophagy, Oxidative stress

## Abstract

**Background:**

To investigate how *Fusobacterium nucleatum* (*Fn*) promotes oxidative stress and mediates proliferation and autophagy in hypopharyngeal squamous cell carcinoma (HPSCC).

**Methods:**

The prognosis for 82 HPSCC cases was retrospectively analyzed. HPSCC cell line FaDu was co-cultured with *Fn*. Knockdown of NUDT1 (shNUDT1 group) was done after observing DNA damage response. CCK8 and tumorigenesis assays for proliferation observation, mitochondria ROS (MitoROS) measurement to examine intracellular oxidative stress, and ELISA to analyze concentration of 8-oxo-2’-deoxyguanosine (8-oxo-dG) in cells. Dual-luciferase reporter assays clarified miR-361-3p connection with NUDT1. Autophagy flow was observed using electron microscopy and related proteins.

**Results:**

*Fn* was highly associated with NUDT1. The shNUDT1 group experienced lower proliferation compared with normal FaDu (NC group) in vivo and in vitro. The shNUDT1 group showed 8-oxo-dG and γH2AX to be elevated. Intracellular ROS decreased in shNUDT1*Fn* group when compared to *Fn* group. Upregulating miR-361-3p could suppress NUDT1 expression and downstream proliferation and autophagy. *Fn* modulated miR-361-3p via OH^−^, which could be proven by H_2_O_2_ assay and N-acetylcysteine.

**Conclusions:**

Higher *Fn* in HPSCC patients suggests poorer prognosis. NUDT1 might affect cell proliferation and autophagy and modulate DNA damage response. The oxidative stress induced miR-361-3p/NUDT1 axis is first introduced in microbiome-carcinoma research.

**Supplementary Information:**

The online version contains supplementary material available at 10.1186/s12885-023-11439-4.

## Background

Hypopharyngeal squamous cell carcinoma (HPSCC) is an aggressive head and neck carcinoma (HNC) with an unfavorable prognosis [1]. The clinical guidelines of NCCN and CSCO have clarified different comprehensive treatments for HPSCC, but which of these is the more optimal approach remains to be investigated [2–4]. Following the inclusion of human papillomavirus (HPV) in pharyngeal carcinoma assessments, researchers gradually identified a link between oral microbiota and the promotion of carcinogenic functions through dysbiosis, colonization, and translocation [5]. Moreover, the consistent composition of biofilms in the oral cavity and adjacent tissues is now considered as a viable predictor of carcinoma prognosis [6–8]. Specifically, *Fusobacterium nucleatum* (*Fn*), has been correlated with a poor prognosis in HNC, notably in oral squamous carcinoma [9], laryngeal squamous carcinoma [10], and tongue carcinoma [11]. To date, *Fn*-carcinoma mechanisms that has been discovered could be summarized as follows: (1) promotion of the Wnt/β-catenin signaling pathway through FadA binding to E-cadherin [12]; (2) cytotoxicity of immune cells such as NK cells and T-cell activity being inhibited [13]; (3) LPS binding to TLR4/MYD88 pathway and mediating downstream NF-κB expression [14]; and (4) the release of Fap2 that binding to Gal-GalNAc ligands [15]. Given this, the next valuable step would be to investigate whether oxidative stress triggered by the microbiome could contribute to HPSCC malignancy. Based on our previous laryngeal carcinoma study, we found that aberrant mismatch repair gene (MMR) could triggered DNA damage response (DDR), suggesting that oxidative stress might also contribute to microbiome-HPSCC malignancy [16]. Overwhelming intracellular reactive oxygen species (ROS), such as ●O_2_^−^ and H_2_O_2_, could be induced by pattern recognition receptors (PRRs) such as lipopolysaccharides-induced inflammatory responses, B or T cell receptor activation, platelet-derived growth factor, and TNF-α upon interaction with pathogen-associated molecular patterns (PAMPs). These ROS, originating from internal metabolism and environmental toxicant exposure, can create a harmful environment for normal cells that is more easily tolerated by cancer cells [17]. Enhanced ROS may instigate autophagy by directing damaged organelles and misfolded proteins to lysosomes for degradation, thereby providing self-energy and macromolecular precursors.

The role of the base excision repair (BER) protein, which is fundamental for the precise and efficient repair of DNA in cells, in instigating autophagy remains a contested topic [18]. Nudix hydrolase 1 (NUDT1), a protein within the BER family, is recognized for its ability to hydrolyze oxidized deoxyribonucleoside triphosphate, serving as a regulator in oxidative stress homeostasis and inflammation [19]. MicroRNAs (miRNAs), a class of non-coding RNA molecules, have been recognized as regulators of approximately 30% of cellular genes through translational suppression or mRNA degradation. They are closely linked to tumor progression and therapeutic responses. Considering the potential crosstalk between certain miRNAs and ROS, it would be valuable to investigate whether any miRNAs can modulate the oxidative stress-mediated NUDT1. Building on this, the present study seeks to address: (1) whether *Fn* can be considered an independent risk factor for HPSCC prognosis and (2) the mechanism how *Fn* triggers HNC malignancy could be modulated by NUDT1.

## Materials and methods

### Patients statement

A total of 111 formalin-fixed paraffin-embedded (FFPE) HPSCC tissue samples were collected by pathologists from June 2015 to March 2018. The inclusion criteria were as follows: (1) pathologist confirmed HPSCC; (2) histories, clinical images, and laboratory data were collected in whole; (3) complete follow-up data, including relapse and complications at least over 3 years. The exclusion criteria were as follows: (1) Cessation of antibiotic intake for less than three months; (2) other concomitant carcinomas; (3) Ongoing therapies targeting chronic inflammation; (4) Data loss. 82 patients were qualified and enrolled. Informed consent was received from all participants before the operation. All FFPE samples were obtained directly from surgical procedures, without prior administration of radiology treatments. The clinical characteristics and prognoses of each person are described in Table 1. The probe of FUS664 (against *Fn*, Cy3-labeled) sequence: 5’-CTTGTAGTTCCGCTACCTC-3’ and EUB338 (against *all-bacteria*, FAM-labeled) sequence: 5’-GCTGCCTCCCGTAGGAGT-3’ were prepared [20]. Fluorescence In Situ Hybridization (FISH) was performed to demonstrate the localization of a bacterial chromosome in cells under fluorescence microscopy. Green fluorescence emanates the FUS664 signal, while red the EUB338 signal, and blue particles are nuclei.

**Table 1:** Clinical characteristics of 82 enrolled HPSCC patients

Characteristics	Number (proportion)
Age (years)	59.39 ± 7.79
Gender(female/male)	1(1.2%)/81(98.8%)
HTN (no/yes)	48(58.5%)/34(41.5%)
DM (no/yes)	74(90.2%)/8(9.8%)
Smoking (no/yes)	26(31.7%)/56(68.3%)
Alcohol (no/yes)	23(28%)/59(72%)
Pathological types (PS/PC/PP)	72(87.8%)/5(6.1%)/5(6.1%)
IC before surgery (no/yes)	78(95.1%)/4(4.9%)
Surgery options (PLPP/TLPP/TLTP)	39(47.6%)/30(36.6%)/13(15.8%)
cT classification (T1-2/T3-4)	42(51.2%)/40(48.8%)
cN classification (N0/N+)	13(15.9%)/69(74.1%)
pT classification (T1-2/T3-4)	39(47.6%)/43(52.4%)
pN classification (N0/N+)	11(13.4%)/71(86.6%)
TNM stage (TNM II/III/IV)	1(1.2%)/18(20.8%)/64(78%)
ENE (- / +)	60(73.2%)/22(26.8%)
Tumor diameters (≤ 4 cm/ > 4 cm)	62(75.6%)/20(24.4%)
Size of lymph node (≤ 3 cm/ > 3 cm)	52(63.4%)/30(36.6%)

**Abbreviation**: HTN, hypertension; DM, Diabetes mellitus; IC, induction chemotherapy; PS, piriform sinus; PR, post cricoid; PP, posterior pharyngeal wall; PLPP, partial laryngectomy and partial pharyngectomy; TLPP, total laryngectomy and partial pharyngectomy; TLTP, total laryngectomy and total pharyngectomy; ENE, extranodal extension.

### Transmission electron microscopy (TEM) and scanning electron microscope (SEM)

For TEM, cells and bacteria were subjected to centrifugation and the resultant precipitate, roughly the size of mung beans, was collected and fixed using TEM-specific fixative. After following specimen processing of TEM protocal and cut into thin slices measuring between 60 and 80 nm thin. The images were captured under a HITACHI microscope (#HT7800).

For SEM, the cells were cultured on slides, fixed with 2.5% glutaraldehyde, followed by specimen processing of SEM protocal. Images were acquired using a ZEISS electron microscope.

### HNSCC cell and bacterial culture

The human HPSCC cell line FaDu (RRID:CVCL_1218) was obtained and authenticated by Cell Bank, Type Culture Collection, Chinese Academy of Sciences (CBTCCCAS). The results of DNA Short Tandem Repeat (STR) profiling analysis was consistent with reference matching from ATCC and DSMZ databases.

FaDu was utilized and cultured in high glucose DMEM medium (HyClone) supplemented with 10% fetal bovine serum (FBS) and 1% penicillin-streptomycin in an incubator (37 °C, 5% CO_2_). The *Fusobacterium nucleatum* strain subsp. nucleatum (ATCC 25586) was grown on Columbia blood agar and cultured overnight at 37 °C under anaerobic conditions. The multiplicity of infection (MOI) is the ratio of bacterial cell to HNSCC cell. The proper ratio used was 100:1. The medium would be replaced by antibiotics-free medium with *Fn* being added to the cell cultures. The FaDu cells and *Fn* were employed in subsequent mycoplasma-free experiments.

### Cell transfection and GFP-mCherry-LC3 puncta formation assays

The control, shRNA lentivirus, control microRNA, and miR-361-3p overexpression adenovirus were assembled by Genomeditech. FaDu cell was transfected with lentivirus and subsequently selected via puromycin. The expression of target genes was verified using RT-PCR and Western-blot.

For GFP-mCherry-LC3 puncta formation assays, commercial GFP-mCherry-LC3 probes product (HanBio, China) was used to label autophagy fluxes across different periods. FaDu cells were transfected with 150 nM GFP-mCherry-LC3 for 24 h, before incubating with *Fn* (MOI = 100) for 2, 6, and 12 h, respectively. The GFP-LC3 puncta and mCherry-LC3 puncta was captured to clarify the autophagy flux [21].

### Proliferation through cell counting kit-8 (CCK-8)

FaDu cells were incubated in 96-well plates with five replicates, and *Fn* (MOI = 50, 100, 250, 500, 1000) was added. The incubation time was set as 1–5 days(d). 10% WST-8 was added to each well and incubated for 1 h. Cell viability was determined at 450 nm absorbance (OD). Chloroquine (#C424619, Aladdin), an autophagy inhibitor, was added to different groups to investigate whether proliferation was inhibited.

### Luciferase assay

Wild-type NUDT1 (NUDT1-wt) and mutant NUDT1 (NUDt1-mut) were generated through cloning the NUDT1-wt 3ʹ-UTR and NUDT1-mut 3ʹ-UTR, then transfected into HEK-293T cells and co-transfected with miR-361 mimic or NC mimic. Dual-Luciferase Reporter Assay System (#E1910, Promega) was employed to treat cells and GloMax 96 Microplate Luminometer was used to define the luciferase activity which was shown as a ratio of firefly luminescence to renilla luminescence.

### Neutral comet assay

The protocol was conducted following CometAssay kit instructions (#4250-050-K, Trevigen) to assess the DDR. The image of comet tails was captured with a fluorescence microscope. Tail DNA (%) and Olive Tail Moment (OTM) were performed using Tritek CometScore software (ver2.0.0.38. https://www.autocomet.com/cometscore/), and OTM was calculated as (Tail.mean - Head.mean)× (Tail%DNA)/100.

### RNA extraction and measurement

Total RNA from cell and tissue samples were extracted through TRIzol reagent. The mRNA or miRNA were reversed transcription to cDNA through PrimeScript RT reagent Kit (#RR047A, Takara) and miScript II RT Kit (#218161, Qiagen). The internal references GAPDH and U6F were set for mRNA and miRNA, respectively. The qPCR Kit for mRNA and miRNA was QuantiNova SYBR Green PCR Kit (#208054, Qiagen) and miScript SYBR Green PCR Kit (#218073, Qiagen), respectively. Comparing expression of mRNA or miRNA through using the -ΔCt or 2^−ΔΔCt^ method. Triplicate samples were verified on an ABI 7500 Real-Time PCR system.

### Detection of *Fn*

The genomic DNA (gDNA) was extracted from fresh human biopsy tissues through the instruction of QIAamp BiOstic Bacteremia (#12240-50, Qiagen). The gene of PGT was used as an internal reference gene [22]. Forward primer of PGT was: 5’-ATCCCCAAAGCACCTGGTTT-3’; reverse primer of PGT was: 5’- AGAGGCCAAGATAGTCCTGGTAA-3’; forward primer of *Fn* was: 5’-CAACCATTACTTTAACTCTACCAT-3’; reverse primer of *Fn* was: 5’-GTTCAGTTGACTTTACAGAAGGAGATTATGTAAAAATC-3’.

### Western blot, IHC, ELISA

For Western-blot, cells and HPSCC tissues were lysed and proteins extracted in 10% PMSF-based RIPA lysis buffer (Beyotime). BCA Assay was used to quantify the concentration of each protein. Each sample was added to 4–20% SDS-PAGE gels and transferred to 0.22 μm PVDF membranes. After the membranes were incubated with the primary and secondary antibodies, enhanced chemiluminescence was used to manifest the concentration under a protein visualizer. The images of the original blot with membrane edge and the explanation of cropped blots would be provided in supplementary materials.

For immunohistochemistry (IHC), FFPE tissues of 82 patients were stained for anti-NUDT1 antibody (#ab197028, Abcam) by following the IHC protocal as previously described. The IOD/Area was interpreted by using Image-Pro Plus 6.0.

For ELISA, samples were detected by the manufacturer’s instructions of 8-oxo-dG ELISA Kit (#D751009, Sangon Biotech).

### MitoROS measurement

Tripled cultured cells were plated in 96-well plates overnight. *Fn* were then added in plates to co-culture with FaDu. 100nM MitoTracker Red CMXRos (#40741ES50, Yeason) was prepared in DMEM medium without FBS. Adding in different experimental designs and incubating at 37 °C, 5% CO_2_, for 30 min. After PBS rinsing samples, the result was observed at wavelength (Ex = 569 nm, Em = 599 nm) in a dark room.

### Xenograph tumor in mice

Six-week-old male BALB/C Slac-nu mice were purchased from Shanghai laboratory animal company, and housed in the Animal Center at the Eye & ENT hospital with the standard temperature range between 22 and 24 °C, the relative ambient humidity range from 50 to 60% and semi-natural daily light/dark cycle. A million well-treated FaDu cells mixed with matrigel (#356234, BD) were subcutaneously injected into the right axillary area of mice. The mice’s weight and tumor size were measured twice a week. Two animal experiments were conducted. In the first experiment, all 14 mice were fed in a month, and the largest tumor closely reached 1.2 cm in diameter. In the other animal experiment investigating knocked down NUDT1, 20 mice were involved and the largest of tumors reached 1.5 cm in diameter. Our strategy of euthanasia was intraperitoneal injection of Zoletil before cervical dislocation. The tumor volume could be calculated with the equation as follows: (Length* Width* Width)/2.

### Statistical analysis

The 21st edition SPSS software (IBM) was used for data processing, and the ninth edition GraphPad Prism was applied for most graphic demonstrations. Pearson’s chi-squared test or Fisher’s exact test was used to analyze clinicopathological parameters associated with stratified *Fn* groups. Receiver operating characteristic (ROC) analysis was performed to assess the optimal cutoff value for predicting HPSCC prognoses. Kaplan-Meier method and log-rank test were depicted for long-term survival status in the categorized group. Two-tailed *p* < 0.05 was considered statistically significant.

## Results

### The prognostic risk factor of *Fn* in HPSCC

**Fig. 1:**
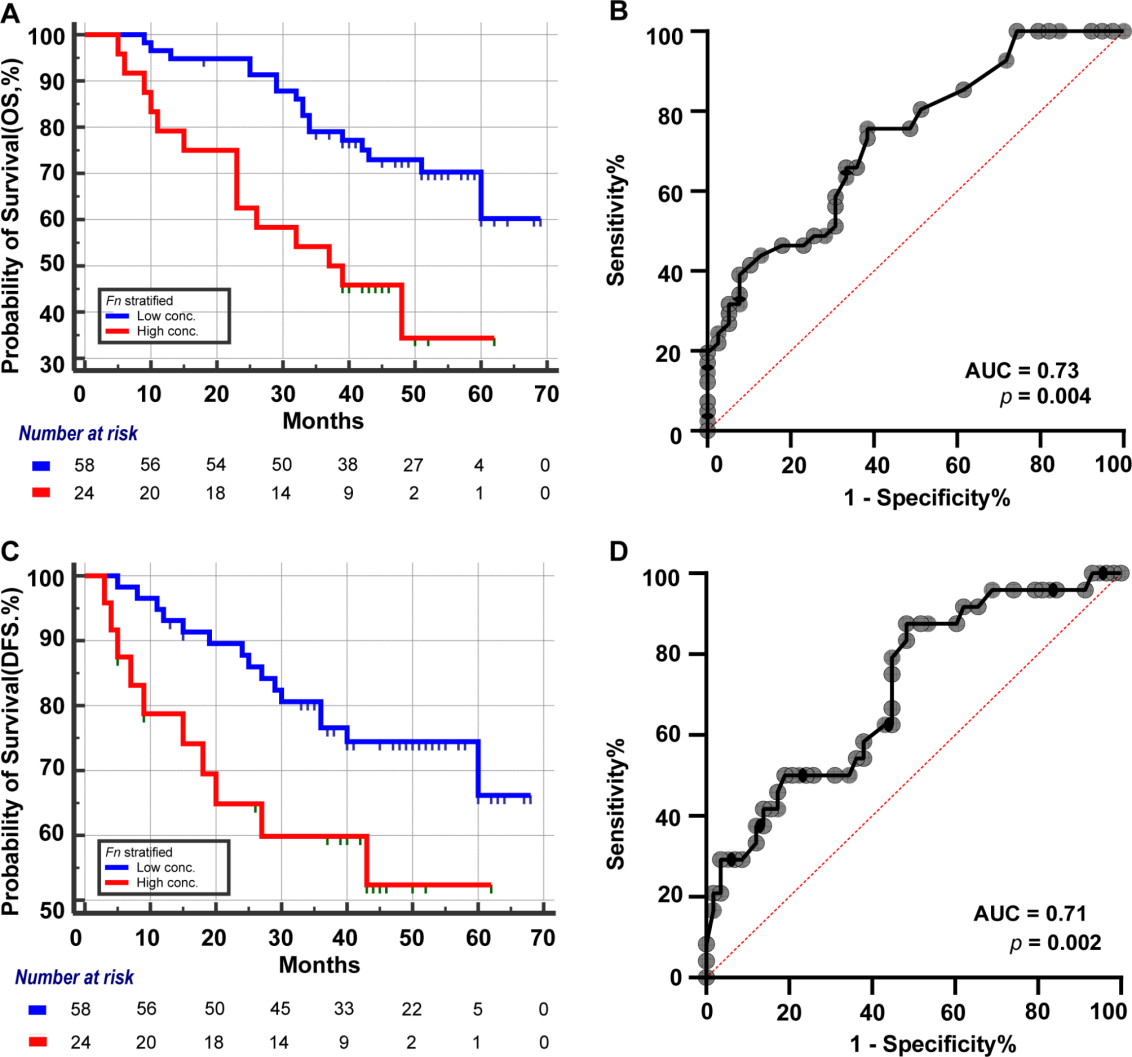
The Kaplan-Meier analyses toward OS, DFS and ROC curve. **(A)** Number at risk for stratified risk group of *Fn* over 5 years OS. **(B)** The ROC curve for OS prediction and the AUC is 0.73. **(C)** Number at risk for stratified *Fn* group over 5 years DFS. **(D)** The ROC curve for DFS prediction and the AUC is 0.71

X-tile (version 3.6.1, Yale University) [23] was applied to select the optimal cut-off point for the *Fn* based on the highest log-rank value and the lowest *p*-value. When *Fn* was set at -ΔCt = -6.7, patients with HPSCC could be divided into a high *Fn* group (-ΔCt ≥ -6.7) and a low *Fn* group (-ΔCt < -6.7). Categorized by high and low *Fn* group, the demographics of 82 HPSCC patients was shown to be even under Pearson’s Chi-squared test (Table S1). The risk factors such as age, hypertension, diabetes mellitus, smoking history, alcohol drinking history, operation options, extranodal extension, tumor diameter size (4 cm), lymph node diameter size (3 cm), T classification, N classification, and TNM staging were enrolled in univariate and multivariate Cox analyses. It was found that a tumor size (4 cm) and *Fn* were independent risk factors in OS (Table 2), while *Fn* was an independent risk factor in DFS (Table 3). The Kaplan-Meier method showed that 83.33% of patients in the low *Fn* group and 59.52% in the high *Fn* group survived till year 3 (Fig. 1A). The AUC for OS prediction with *Fn* was 0.73 (Fig. 1B). The Kaplan-Meier method showed that 75.09% of patients in the low *Fn* group and 59.86% in the high *Fn* group survived without disease recurrence till year 3 (Fig. 1C). The AUC for DFS prediction with *Fn* was 0.71 (Fig. 1D).

**Table 2:** Univariate and Multivariate analysis of Overall Survival (OS)

Characteristics	OS (HR)	*P* value (95% CI)		OS(HR)	*P* value (95% CI)
**Age (year)** $<$ 60 $\ge$ 60	1 1.468	0.289 (0.722–2.981)	NA		NA
**HTN** No Yes	1 0.929	0.842 (0.450–1.916)	NA		NA
**DM** No Yes	1 1.063	0.920 (0.322–3.506)	NA		NA
**Smoke History** No Yes	1 0.682	0.299 (0.332–1.404)	NA		NA
**Drinking History** No Yes	1 1.328	0.491 (0.593-2-971)	NA		NA
**Vascular Embolus** No Yes	1 2.358	0.080 (0.903–6.154)	NA		NA
**Extracapsular spreads** No Yes	1 1.370	0.414 (0.643–2.917)	NA		NA
**Size of tumor (cm)** $\le$ 4 $>$ 4	1 2.463	0.015* (1.192–5.086)	1 2.321		0.024* (1.118–4.819)
**Size of lymph node (cm)** $\le$ 3 $>$ 3	1 1.229	0.578 (0.594–2.545)	NA		NA
**Clinical Stage (**TNM**)** II-IVA IVB	1 1.049	0.908 (0.468–2.351)	NA		NA
**Tumor classification** T1-T2 T3-T4	1 1.715	0.144 (0.831–3.540)	NA		NA
**Node classification** N0 N+	1 1.152	0.792 (0.403–3.294)	NA		NA
***Fn*** Low event High event	1 2.925	0.003* (1.429–5.986)	1 2.780		0.005* (1.359–5.688)
**Differentiation Grade** Poor Well and moderate	1.059 1	0.915 (0.367–3.062)	NA		NA

Statistics were tested under Cox proportional regression method. *p < 0.05 means statistical significance with bold marker. Abbreviation: HTN, hypertension; DM, Diabetes mellitus

**Table 3:** Univariate and Multivariate analysis of Overall Survival (DFS)

Characteristics	DFS (HR)	*P* value (95% CI)	DFS(HR)	*P* value (95% CI)
**Age (year)** $<$ 60 $\ge$ 60	1 1.127	0.749 (0.544–2.335)	NA	NA
**HTN** No Yes	1 1.187	0.646 (0.571–2.470)	NA	NA
**DM** No Yes	1 0.325	0.269 (0.044–2.390)	NA	NA
**Smoke History** No Yes	1 0.778	0.514 (0.366–1.655)	NA	NA
**Drinking History** No Yes	1 2.228	0.104 (0.849–5.837)	NA	NA
**Vascular Embolus** No Yes	1 2.143	0.158 (0.743–6.180)	NA	NA
**Extracapsular spreads** No Yes	1 1.079	0.856 (0.476–2.442)	NA	NA
**Size of tumor (cm)** $\le$ 4 $>$ 4	1 3.099	0.003* (1.469–6.537)	1 2.306	0.080 (0.906–5.868)
**Size of lymph node (cm)** $\le$ 3 $>$ 3	1 1.163	0.694 (0.549–2.464)	NA	NA
**Clinical Stage (**TNM**)** II-III IVA-IVB	1 1.109	0.821 (0.451–2.725)	NA	NA
**Tumor classification** T1-T2 T3-T4	1 2.155	0.050* (0.998–4.652)	1 1.488	0.415 (0.572–3.870)
**Node classification** N0 N+	1 1.059	0.915 (0.368–3.045)	NA	NA
***Fn*** Low event High event	1 2.686	0.009* (1.287–5.606)	1 2.602	0.011* (1.242–5.452)
**Differentiation Grade** Poor Well and moderate	1.265 1	0.703 (0.367–4.238)	NA	NA

Statistics were tested under Cox proportional regression method. *p < 0.05 means statistical significance with bold marker. Abbreviation: HTN, hypertension; DM, Diabetes mellitus

### The appearance of *Fn* in HPSCC tissue and their ability to boost proliferation

**Fig. 2:**
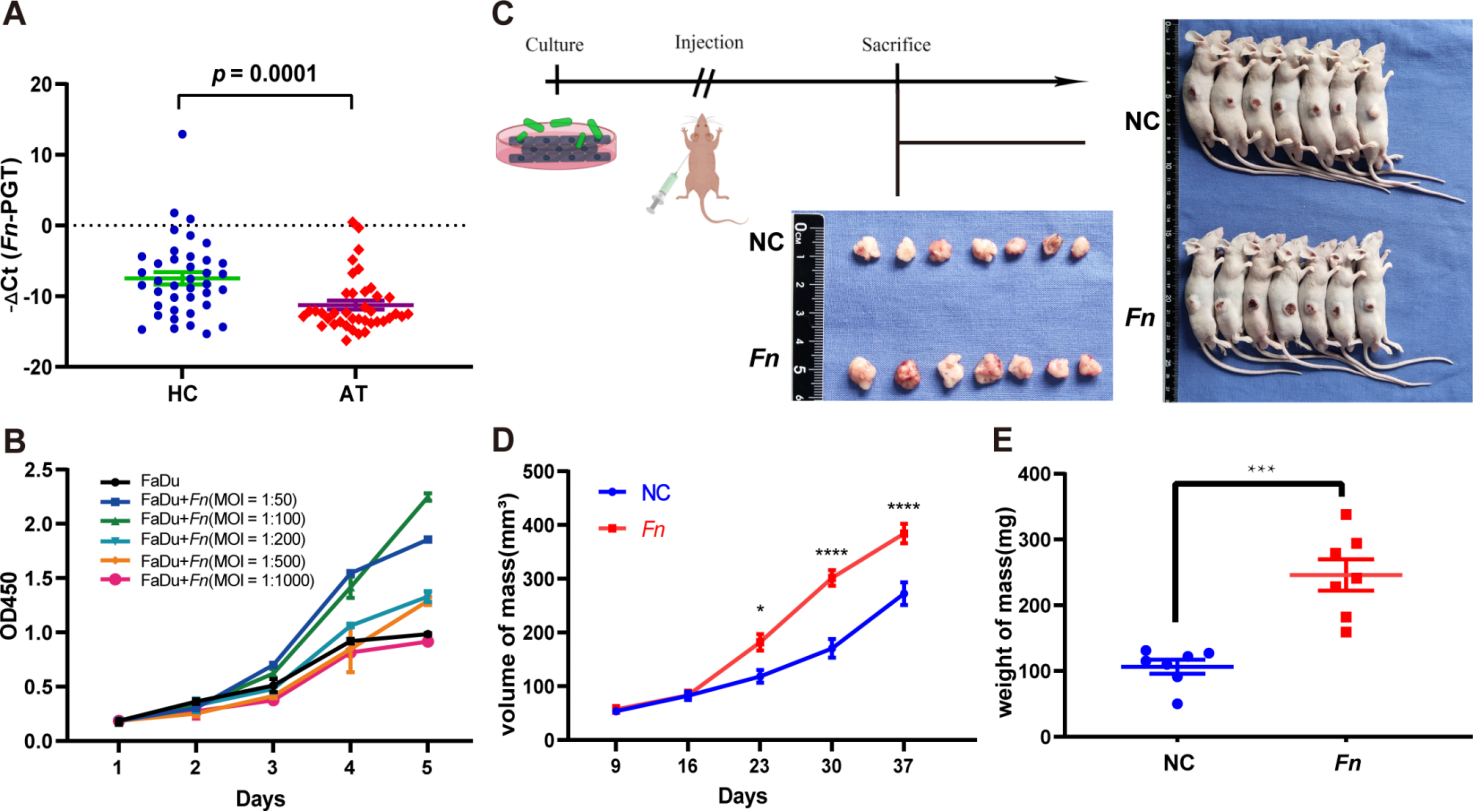
Distribution of *Fn* and proliferation in *Fn*-HPSCC in vitro and in vivo. **(A)** The absolute abundance of *Fn* was detected higher in hypopharyngeal carcinoma tissue (HC), when compared with adjacent tissue (AT). **(B)** The MOI of *Fn* at 100 had a better ability to trigger the proliferaton of FaDu. **(C)** The time flow of tumorigenesis assays and appearance of sacrificed nude mice and mass. **(D)**The volume of mass. **(E)** The weight of mass

The distribution of all-bacteria and *Fn* on paraffin samples were confirmed to attach to hypopharyngeal squamous carcinoma tissues (Fig S1A-C). To exclude possible manipulation bias in paraffin samples, 37 pairs of fresh hypopharyngeal carcinoma tissues and their paracancerous tissues were cross-validated (Fig. 2A). SEM revealed the presence of microvillous structures on the surface of FaDu cells, which were abundant and morphologically full. After co-culture with *Fn*, the microvillous structures on carcinoma cells became wrinkled. The morphology of *Fn* presents uneven length and partial dissolution of the capsule structure. (Fig S1D-F).

The proliferation ability of FaDu cells significantly promoted tumor cell proliferation by adding *Fn* with MOI = 50–250, among which *Fn* with MOI = 100 had a higher effect. Thus, we used MOI = 100 as the standard in the following study. Adding *Fn* with MOI > 500 had an inhibitory effect (Fig. 2B). Tumorigenesis assays in nude mice were also performed to demonstrate that size and weight of mice were significantly larger in *Fn* group (Fig. 2C-E).

.

### *Fn* facilitates oxidative stress and autophagy in HPSCC

Chronic inflammation could trigger ROS rising in epithelial cell and enhance mutagenicity during replication, which might stimulate various important signaling pathways in carcinogenesis [24]. It is still questionable whether *Fn*, a resident bacterium in oral cavity, can induce enough oxidative stress in carcinoma. MitoROS measurement revealed that the upregulation of *Fn* content, the stress of intracellular oxidative stress was significantly increased, and the peak is achieved when *Fn* (MOI = 250) was reached.

**Fig. 3:**
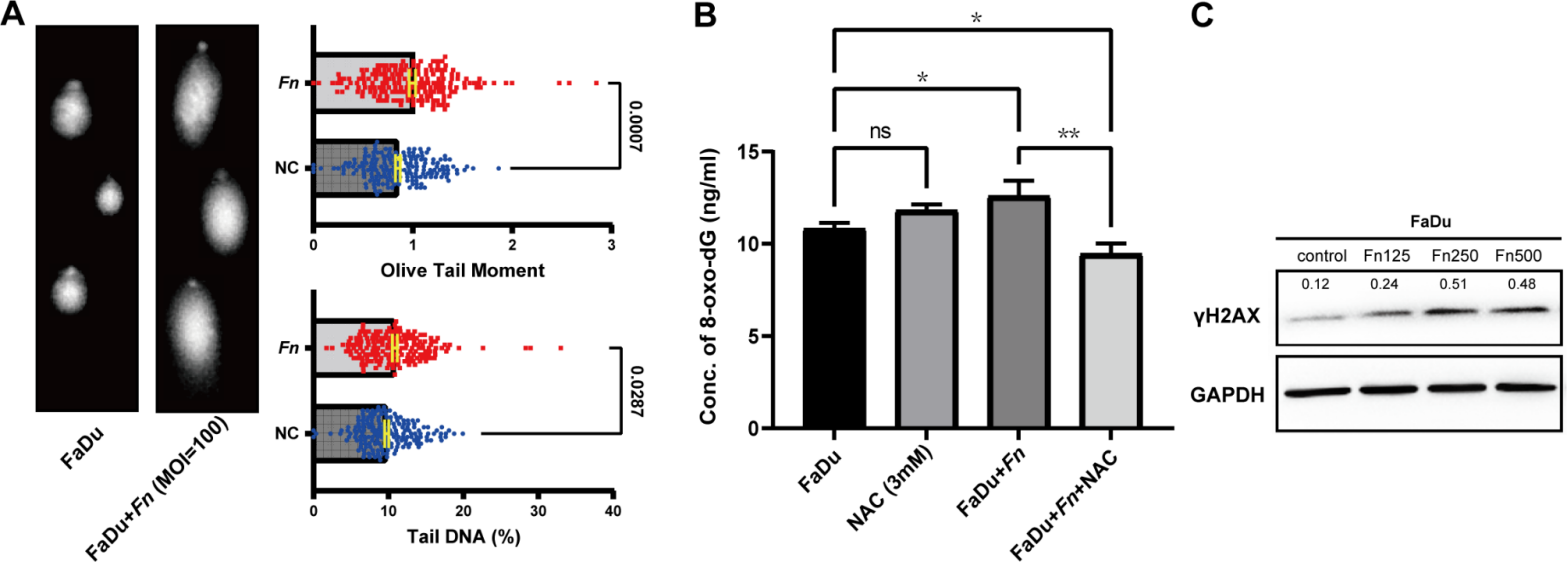
FaDu was found DNA damage response when cultured with *Fn*. **(A)** The tail of DNA fragment, the olive tail moment (OTM) and tail DNA (%) found greater in *Fn* group compared with NC group, showing the significant difference. **(B)** ELISA proved that *Fn* facilitate 8-oxo-dG production, indicating oxidative stress join the malignancy. **(C)** Western-blot showed γH2AX increased after adding different MOI of *Fn*

Elevated ROS could trigger DDR and abnormal metabolism of nucleotide pools. Comet electrophoresis suggests that *Fn* could induce severe DDR, where both OTM and tail DNA were significantly elevated (Fig. 3A). 8-oxo-dG, the main metabolite of glycosylated guanosine with G:C to T:A transversions, was also used to assess the extent of oxidative stress. Carcinoma cells infected by *Fn*, yielded upregulation of 8-oxo-dG. After blocking with N-acetylcysteine (NAC), the status of oxidative stress was found to be downregulated (Fig. 3B). γH2AX was also able to recruit and localize DNA repair proteins to form foci with double-stranded breaks (DSBs) in a 1:1 manner, which has been used as a biomarker to signal DNA damage [25]. Western-blot suggested that γH2AX gradually increased after adding different gradients of *Fn* (Fig. 3C).

TEM showed that the overall morphology of normal FaDu cells was round and regular. The surrounding microvilli (Mv) were locally distributed with some being swollen and round; the nucleus was irregularly shaped, locally concave, and centered. Mitochondria (M) were swollen, larger in volume, with uneven matrix, and cristae broken. After *Fn* infected FaDu cells, the carcinoma cells became shuttle-shaped. The nucleus became oval-shaped. Mitochondria were abundant in number and increased in volume with bulging membrane. Individual bacteria particles (*Fn*) could be seen connecting near the extracellular microvilli.

**Fig. 4:**
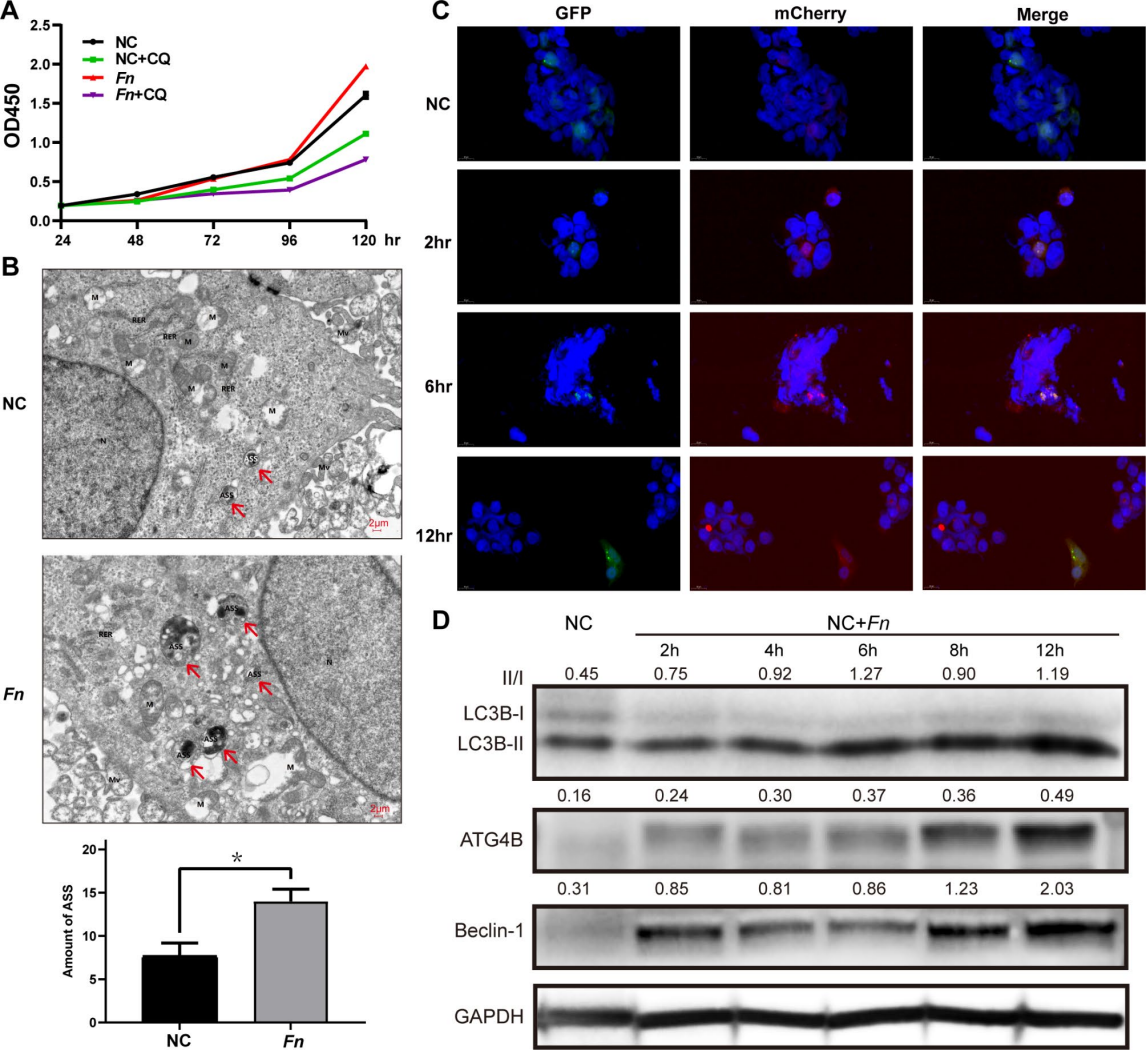
Autophagy flux in *Fn* cocultured FaDu. **(A)** The proliferation of *Fn* group was inhibited severely by chloroquine (CQ). **(B)** In TEM, ASS was found rising in *Fn* group, when compared to NC group. **(C)** The proportion of mCherry-LC3 puncta formation increase indicates activation of autophagy flow. **(D)** Western-blot proved that autophagy-related proteins were rising in the period of 2–12 h after adding *Fn*. Red arrow indicated ASS.

Chloroquine (CQ) could significantly inhibit proliferation of FaDu + *Fn* group (*Fn* group), showing that autophagy might be functional in this microbiome-cancer progress (Fig. 4A). A higher amount of autolysosomes (ASS) could be discovered in the *Fn* group compared with NC group (Fig. 4B). GFP-mCherry-LC3 puncta formation assay was applied to demonstrate the dynamic change from autophagosome to ASS, in which GFP-LC3 protein could be eradicated when meeting with lysosome and mCherry-LC3 left. NC group have a low degree of red puncta. After adding *Fn* to FaDu for two to 12 h, the proportion increased, suggesting that *Fn* facilitates the process of autophagic flow (Fig. 4C). Western-blot showed the hub autophagy proteins (Beclin-1, LC3B, and ATG4B) were significantly upregulated in the period of *Fn* incubation time of 2 h (h), 4 h, 6 h, 8 h, and 12 h for autophagic flow, compared with NC (Fig. 4D).

### NUDT1 knockdown was associated with proliferation decreasing

**Fig. 5:**
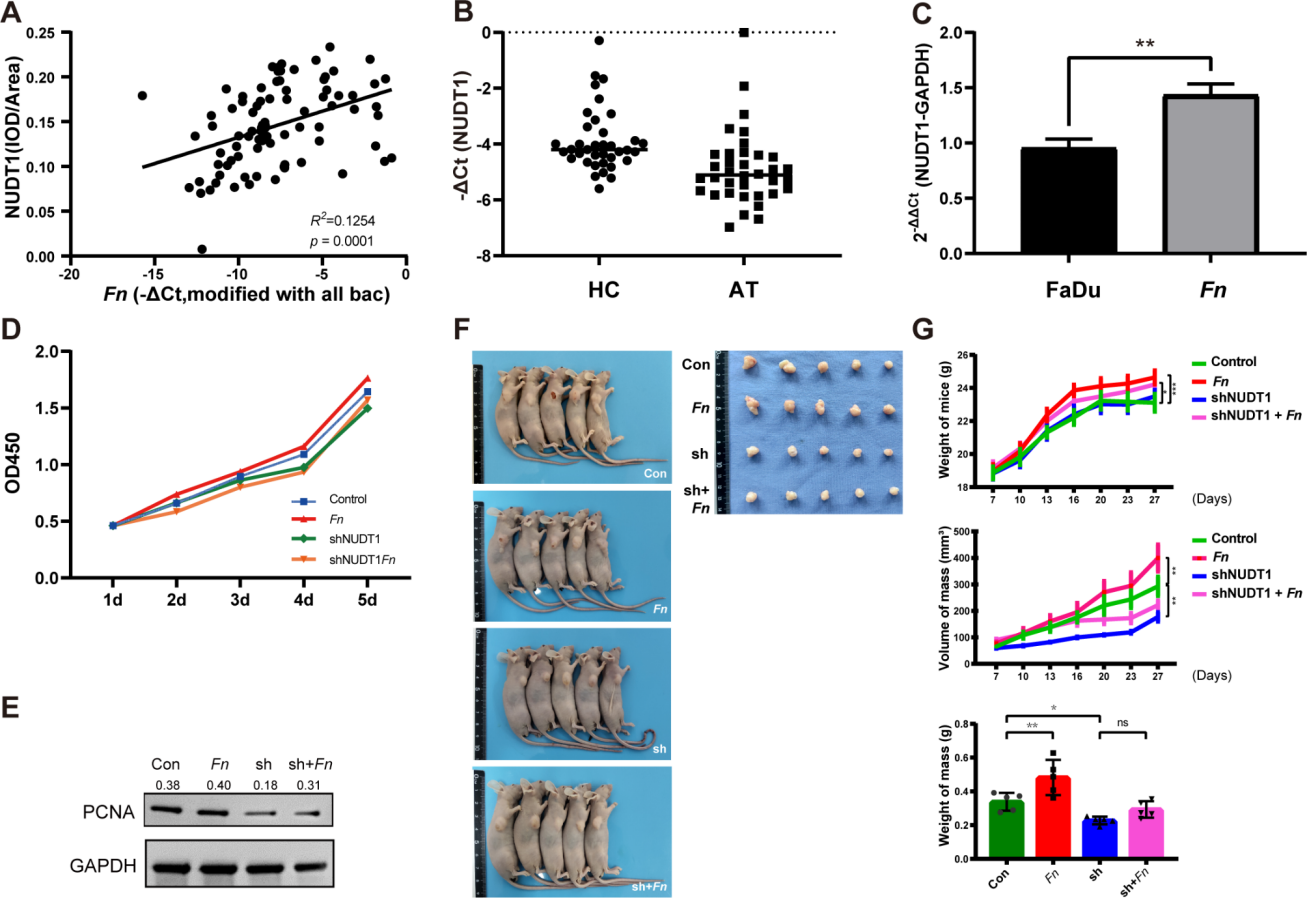
Distribution of NUDT1 and how NUDT1 affects proliferation in vitro and in vivo. **(A)** High *Fn* group showed higher NUDT1 when compared to low *Fn* group. **(B)** HC group had higher NUDT1 expression compared to AT group. **(C)***Fn* could stimulate NUDT1 expression in FaDu cells. **(D)** CCK-8 disclosed that downregulating NUDT1 (shNUDT1 group) would weaken the cell proliferation. **(E)** Western-blot proved that PCNA was found downregulation in shNUDT1 as well. The blot have been cropped and edited to better display groupings within the same gel. **(F)** In vivo, the appearance of mice and mass was displayed. **(G)** The weight of mass, volume of mass and weight of mice showed decline in shNUDT1 group

NUDT1 expression was significantly increased when *Fn* was elevated, showing a positive correlation between the two indices (Fig. 5A). A total of 37 paired tissue of hypopharyngeal carcinoma (HC) and their adjacent tissue (AT) were validated again with higher NUDT1 mRNA present in HC tissue (Fig. 5B). Together with the western-blot result, HC tissue had statistically higher protein levels of NUDT1 compared to AT group (Fig S3A-B). *Fn* could boost intracellular NUDT1 in FaDu cells (Fig. 5C). The NUDT1 knockdown group was built (shNUDT1 group). Both CCK-8 assays and EdU assay suggested that decreasing NUDT1 could inhibit the proliferation of carcinoma cells, even when infected with *Fn* (Fig. 5D, Fig S3C). The PCNA expression also downregulated when NUDT1 was knocked down (Fig. 5E). In vivo tumorigenesis assays, the body weight, tumor volume and tumor weight of mice in shNUDT1 group and *Fn*-infected shNUDT1 (shNUDT1*Fn*) group were significantly decreased compared with FaDu group with statistical differences (Fig. 5F-G).

### Knockdown of NUDT1 boosts 8-oxo-dG elevation, but inhibits intracellular ROS status and autophagy genes

**Fig. 6:**
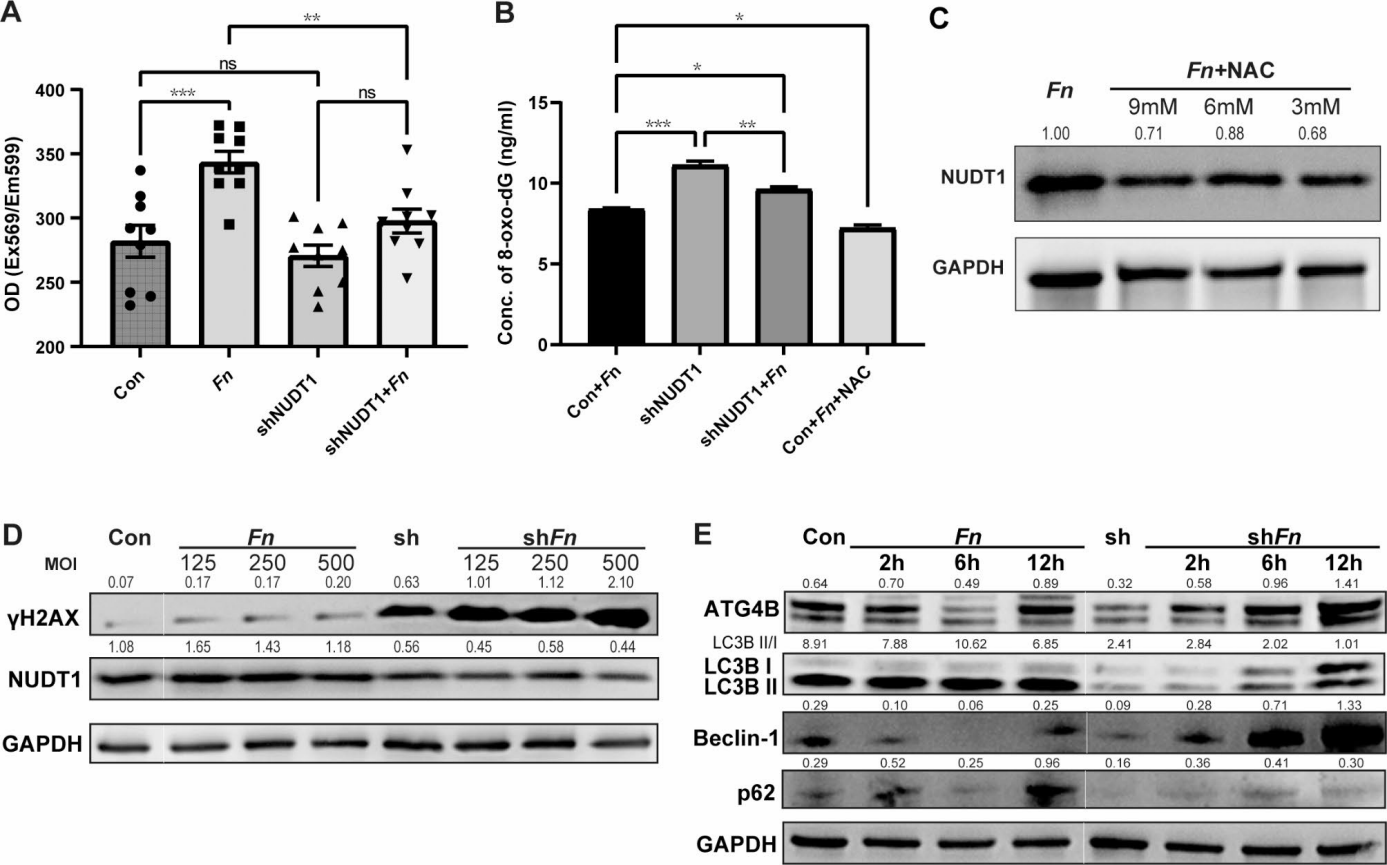
How downregulation of NUDT1 affects ROS status, 8-oxo-dG and autophagy. **(A)** shNUDT1 group did not show significant variation in intracellular ROS when compared to the control group. However, shNUDT1 group could modulate *Fn*-produced ROS. **(B)** shNUDT1 group had higher 8-oxo-dG, when compared to *Fn* group. After ROS inhibited by NAC, 8-oxo-dG was decreased as well. **(C)** Western-blot proved that NUDT1 could be modulated by *Fn* triggered ROS. **(D)** shNDUT1 group presents higher DDR, which showed that NUDT1 could repair DDR at some content. **(E)** Autophagy-related protein was downregulated in shNUDT1. LC3B showed autophagy stagnant when shNUDT1 group co-cultured with *Fn* in the period of 2–12 h. The blot have been cropped and edited to better display groupings within the same gel

Compared to the control group, shNUDT1 group did not show a significant increase in intracellular ROS. Intriguingly, shNUDT1*Fn* group present lower ROS production compared to *Fn* group (Fig. 6A), suggesting that NUDT1 could modulate intracellular ROS status. Compared with *Fn* group, shNUDT1 group presented higher 8-oxo-dG, which could be rescued by NAC (Fig. 6B). The expression of NUDT1 could be inhibited by downregulation of intracellular ROS (Fig. 6C). The rising concentration gradient of *Fn* could significantly enhance expression γH2AX in the shNUDT1 group when compared to the control group (Fig. 6D). *Fn*-enhanced autophagy in hypopharyngeal squamous carcinoma could be turned over by decreasing NUDT1. We found that ATG4B, LC3BII, Beclin-1, and p62 were significantly downregulated in shNUDT1 group, when compared to control group. It was found that LC3B presented obviously stagnant even after incubating with *Fn* along with increasing time. Both LC3BII/I ratio and LC3BII were much lower in the shNUDT1 group and shNUDT1*Fn* group (Fig. 6E).

### MiR-361-3p could directly modulate NUDT1 expression

**Fig. 7:**
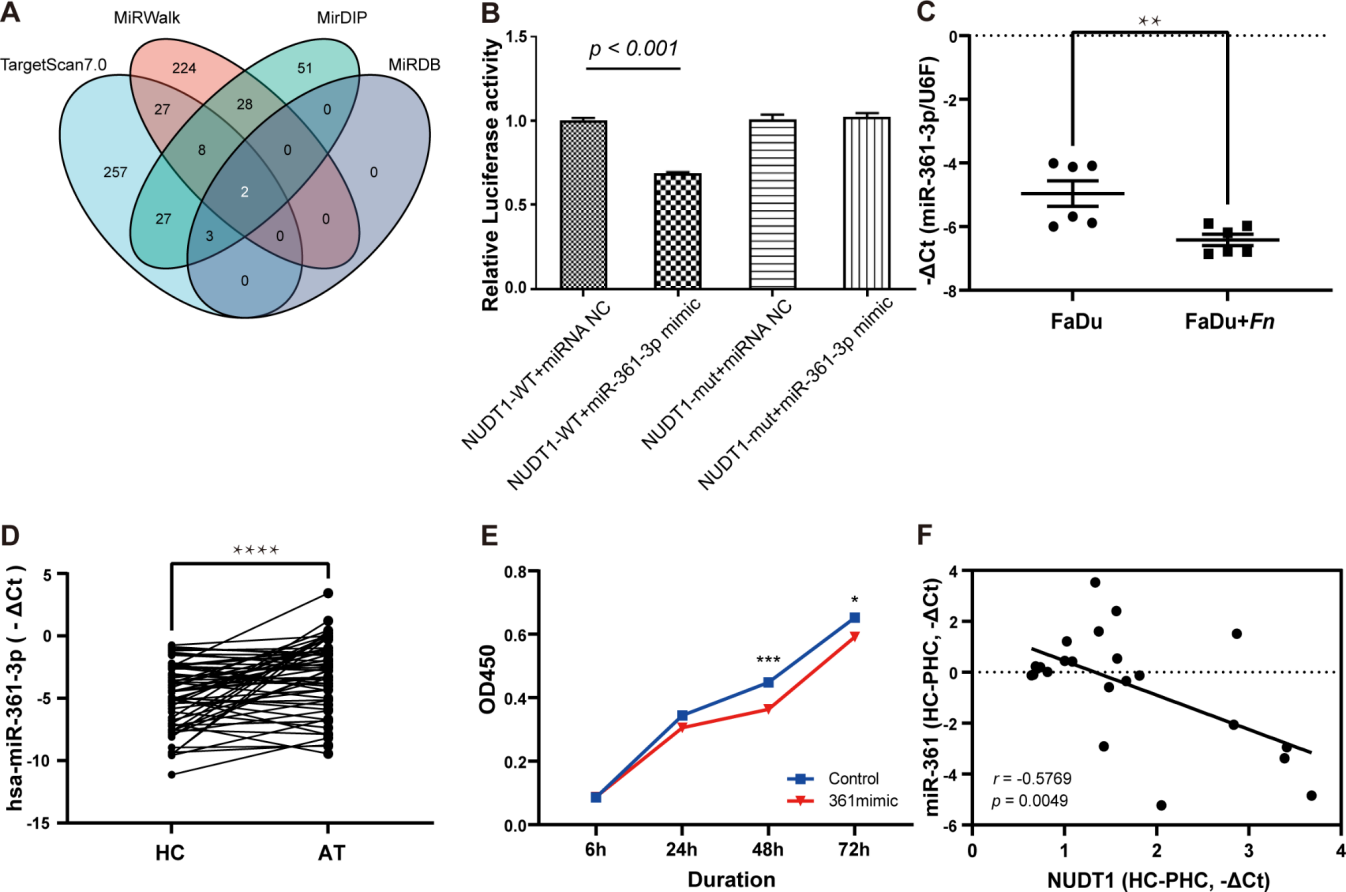
The miR-361-3p could be strongly connected with NUDT1. **(A)** 4 miRNA-gene databases were collected to analyze and found miR-361-3p has a better connection with NUDT1. **(B)**The dual-luciferase assay proved that miR-361-3p could straightly connect with NUDT1. **(C)** MiR-361-3p was found to be decreased in NC group but **(D)** increased in AT group, which implies that miR-361-3p might be a protective agent. **(E)** MiR-361-3p mimic had a weaker proliferation ability, compared to the control group. **(F)** MiR-361-3p presented a negative correlation with NUDT1.

TargetScan (https://www.targetscan.org/vert_80/), miRWalk (http://mirwalk.umm.uni-heidelberg.de/), mirDIP (http://ophid.utoronto.ca/mirDIP/), and miRDB (http://www.mirdb.org/) were used to predict microRNA against NUDT1. The miR-361-3p and miR-145-5p were finally screened out. Among them, miR-361-3p was selected for its stronger binding ability and higher target score, to be used in subsequent experiments (Fig. 7A). The dual-luciferase assay confirmed that NUDT1-wt instead of NUDT1-mut group could be significantly reduced by miR-361-3p overexpression, showing a direct connection (Fig. 7B). *Fn* triggered miR-361-3p downregulation in FaDu (Fig. 7C) and higher levels of miR-361-3p present in AT group instead of HC group (Fig. 7D). Overexpressed miR-361-3p was found to inhibit FaDu cell proliferation (Fig. 7E). The correlation between miR-361-3p and NUDT1 in patients presented a significant negative correlation (Fig. 7F).

### MiR-361-3p influenced by *Fn*-ROS could modulate LC3B expression

**Fig. 8:**
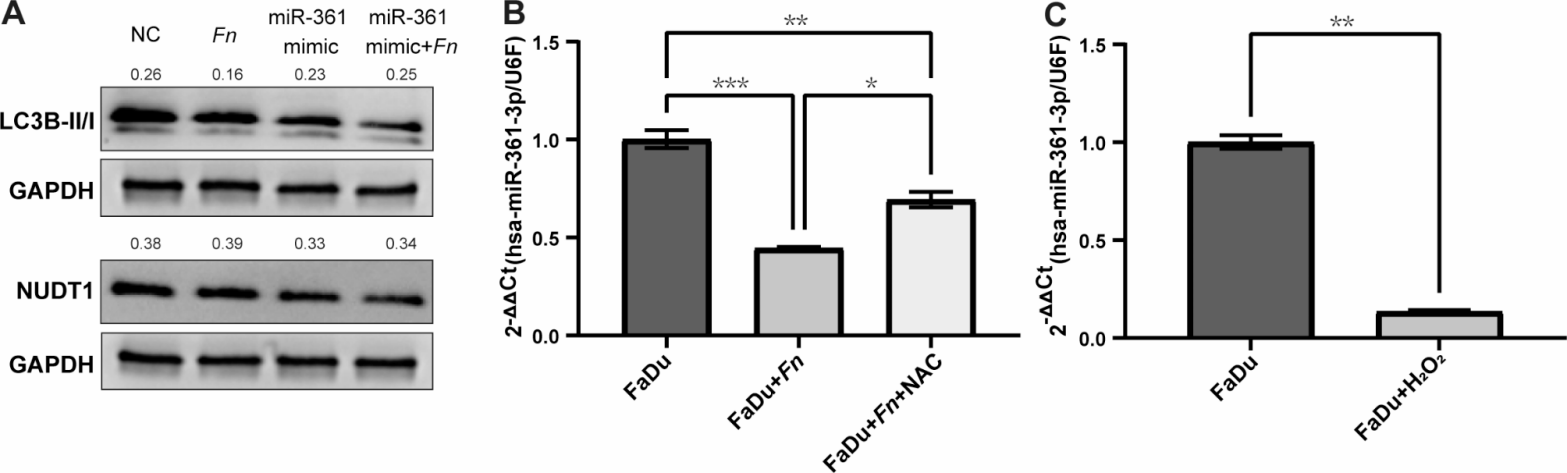
Overexpression of miR-361-3p could inhibit autophagy, which could be modulated by ROS. **(A)** MiR-361-3p mimic could decrease the expression of NUDT1 and also LC3B, compared to NC group. **(B)** qPCR validated that ROS might be the factor directly inhibiting the miR-361-3p. **(C)** H_2_O_2_ assay indicated ROS showed the same pattern as *Fn*-induced oxidative stress, which could further inhibit miR-361-3p. *p < 0.05

Since knockdown of NUDT1 was found to mainly interfere with LC3B of the autophagic pathway in the previous study, further validation revealed that LC3BII and LC3BII/I ratio were also downregulated after upregulating miR-361-3p (Fig. 8A). NAC could rescue expression of *Fn* inhibiting miR-361-3p (Fig. 8B), concluding that *Fn* does affect the downstream miR-361-3p/NUDT1 axis via ROS. H_2_O_2_ assay demonstrated that elevated intracellular oxidative stress inhibited miR-361-3p (Fig. 8C).

## Discussion

The association between microbiota and the progression of carcinoma is gradually being recognized, and currently, approximately 13% of human carcinoma is proven to be the consequence of microbial infection [26]. *Fn*, a causative agent of periodontal diseases, has a coaggregation ability to form an oral biofilm, potentially affecting the intracellular oxidative TME. Environmental insults, due to their ubiquity and complexity, are closely linked with a multitude of diseases. Oxidative stress and inflammation, as well as alterations in microbiota communities, are two of the eight hallmarks of environmental insults [27]. Prior research has provided evidence that microbiota can incite intracellular oxidative stress. The ‘multiple hit’ hypothesis posits that oxidative stress can promote the progression of non-alcoholic steatohepatitis, a significant precursor to liver cancer development [28]. The present study investigated whether *Fn* facilitates HPSCC malignancy through mechanism of oxidative stress and affirmed NUDT1, a BER gene, could affect autophagy and proliferation through this *Fn*-HPSCC mechanism.

The oropharynx microbiota in HPSCC patients exhibits dysbiosis characterized by reduced diversity and abundance [29]. *Fusobacterium* is a resident bacterium in oral cavity. Irregular increases in *Fn*, potentially resulting from unnoticed personal habit, are considered an independent risk factor for carcinoma progression. According to our previous absolute quantification profiling on HPSCC, a similar biofilm exists in the oropharynx and hypopharynx areas, where *Fusobacterium* is linked to more advanced T classification and TNM staging [30]. In our retrospective study, HPSCC patients with higher *Fn* group presented worse prognosis. In vitro and in vivo experiments showed that *Fn* could stimulate FaDu proliferation and S-phase cell cycle arrest, suggesting increased intracellular DNA replication or DNA damage. Oxidative stress-induced DDR might account for this phenomenon (Fig S4A).

Excess oxidative stress is cytotoxic to both carcinoma and normal cells, potentially damaging protein, nucleic acids, and lipid synthesis [31], leading to cell apoptosis, senescence and ferroptosis [32, 33]. However, carcinoma cells may display greater sensitivity and tolerance to enhance ROS by triggering antioxidant defense systems such as ascorbic acid, glutathione, ROS-interacting enzymes (SOD, peroxidases and catalases) [34]. Higher oxidative stress is often found in most carcinoma studies, leading to genetic polymorphisms that adapt to the formidable microenvironment, thereby facilitating cell survival and progression [35, 36]. After co-culturing with *Fn*, DNA DSBs and trailing phenomenon increased, suggesting that the antioxidant defense system might be ineffective in modulating intracellular oxidative stress. Further evidence is that 8-oxo-dG increased in *Fn* group but could be inhibited when NAC eradicated intracellular ROS, suggesting that ROS could play a role in *Fn*-carcinoma mechanism.

Elevated ROS can also stimulate cell autophagy, which can play a cytoprotective role in maintaining the cell microenvironment [37]. However, the dichotomous roles of autophagy should be acknowledged, as it can both inhibit carcinoma in the early stages and facilitate progression in the advanced stages by (1) degrading tumor suppressors in tumor cells, thus promoting multi-drug and radiation resistance, (2) inhibiting cell apoptosis, and (3) assisting carcinoma cells in evading the immune system [38, 39]. To determine the function of autophagy in HPSCC progression, CQ was used to suppress in *Fn*-induced carcinoma proliferation. Further, We found that *Fn* reactivated the blockage of autophagy flow (Fig S2) and triggered formation of ASS, suggesting that autophagy could potentially contribute to HPSCC survival [40–42]. The hub autophagy proteins such as Beclin-1, ATG4B, LC3BII/I ratio are upregulated in HPSCC with *Fn*. ROS have been shown to induce autophagy-related gene expression, such as ATG4/LC3 and Beclin1, or ATG7 in autolysosomal fusion through various mechanisms, and can proceed to inhibit P13K-Akt-mTOR, MAPK, JNK and other signaling pathways. Given that most HPSCC patients are diagnosed at an advanced stage at their first medical visit, autophagy is hypothesized to aid carcinoma cell establish suitable TME and boost malignant progression.

Previous studies have demonstrated that NUDT1 is more crucial for tumor cells than nomal cell to alleviate high intracellular ROS levels, particularly 8-oxo-dG. It has also been revealed that NUDT1 may serve as a predictor of poor prognosis in several tumor types such as gastric carcinoma [43], colon carcinoma [44], and oral squamous carcinoma [45]. However, the mechanism of NUDT1 activation and its potential role in initiating autophagy remained unclear. One study confirmed that NUDT1 could facilitate the mutation of the *RAS*, thereby damaging the tumor DNA structure [46]. Another study suggested that NUDT1 upregulates p53 and p21, leading to increased apoptosis of tumor cells [47]. Our study demonstrated a positive correlation between the variation of NUDT1 and *Fn* infection. When NUDT1 was knocked down (the shNUDT1 group), there was a marked inhibition of cell proliferation, observed both in vitro and in vivo. Notably, the ROS status did not show a significant difference between the shNUDT1 group and the control group. However, the shNUDT1*Fn* group exhibited lower ROS levels and higher levels of 8-oxo-dG in comparison to the *Fn* group. This suggests that the downregulation of NUDT1 may diminish the sensitivity to ROS in carcinoma cells. Additionally, the shNUDT1*Fn* group displayed pronounced cell apoptosis when juxtaposed with the control group (Fig S4B). Collectively, these findings suggest a potential role of NUDT1 in autophagy, given the known capability of autophagy to reduce apoptosis and initiate antioxidant processes. The levels of ATG4B, LC3BII, Beclin-1 were suppressed in the shNUDT1 group, and the conversion from LC3BI to LC3BII showed stagnant in the shNUDT1*Fn* group. This further supports our hypothesis that NUDT1 plays a role in autophagy.

Mitochondria are the primary source of ROS, converting between 0.2 and 2% of oxygen into superoxide anions (●O_2_^−^) in the body. H_2_O_2_ is primarily produced by manganese superoxide dismutase (MnSOD), and it can penetrate the mitochondrial membrane, functioning as a second messenger which activates other molecular signaling cascades such as AP-1, HiF-1α and others [48]. Therefore, the direct impact of oxidative stress status on NUDT1 expression has garnered our attention. Recently, a group of ROS sensitive miRNAs termed ROSmiRs, has been discovered and can result in downstream biological responses. It was the first time that we screened out and found that miR-361-3p had high affinity with NUDT1. In alignment with prior research, miR-361-3p is recognized as a tumor suppressor gene, demonstrated by its decreased expression in the HC group relative to the AT group. We hypothesize that the reduced expression of miR-361-3p may be attributable to elevated intracellular oxidative stress. Overexpression of miR-361-3p has been observed to inhibit FaDu cell proliferation and LC3B formation. Notably, NAC was able to mitigate this suppression. The direct stimulation of H_2_O_2_ instead of *Fn* triggered ROS, which reaffirms our discovery concerning the existence of the oxidative stress mediated miR-361-3p/NUDT1 axis.

Oxidative stress plays a crucial role in the initiation and progression of carcinoma. Here we highlighted the important role of oxidative stress in the mechanism of *Fn*-carcinoma and how NUDT1-related autophagy may play a role in the progression of HPSCC. The potential use of NUDT1 inhibitors in the comprehensive treatment of HPSCC could be noted in the future.

## Conclusion

*Fn* could be considered an independent risk factor in HPSCC, suggesting worse prognosis in high-*Fn* patients. NUDT1 was found to balance intracellular oxidative stress and autophagy, which facilitating cell proliferation. The oxidative stress mediated miR-361-3p/NUDT1 axis was introduced and validated in this microbiome-carcinoma research.

### Electronic supplementary material

Below is the link to the electronic supplementary material.

Supplementary Material 1

Supplementary Material 2

Supplementary Material 3

Supplementary Material 4

Supplementary Material 5

Supplementary Material 6

## Data Availability

All data generated or analysed during this study are included in this published article.
