# Supplementary material for: Fusobacterium nucleatum facilitates proliferation and autophagy by activating miR-361-3p/NUDT1 axis through oxidative stress in hypopharyngeal squamous cell carcinoma
Source: BMC Cancer. 2023 Oct 17;23:990. doi: 10.1186/s12885-023-11439-4 (PMC10580517; doi:10.1186/s12885-023-11439-4)

Fig S1. The distribution of *Fn* in HPSCC patients and FaDu cells. (A) FISH showed that *Fn* exists in carcinoma tissue. (B) FISH showed that *all-bacterium* exists in carcinoma tissue. (C) Merged. (D) The surface of FaDu cell in SEM. (E) Microvillus structure could be discovered in normal FaDu surface. (F) *Fn* could intertwine with these microvillus structures.

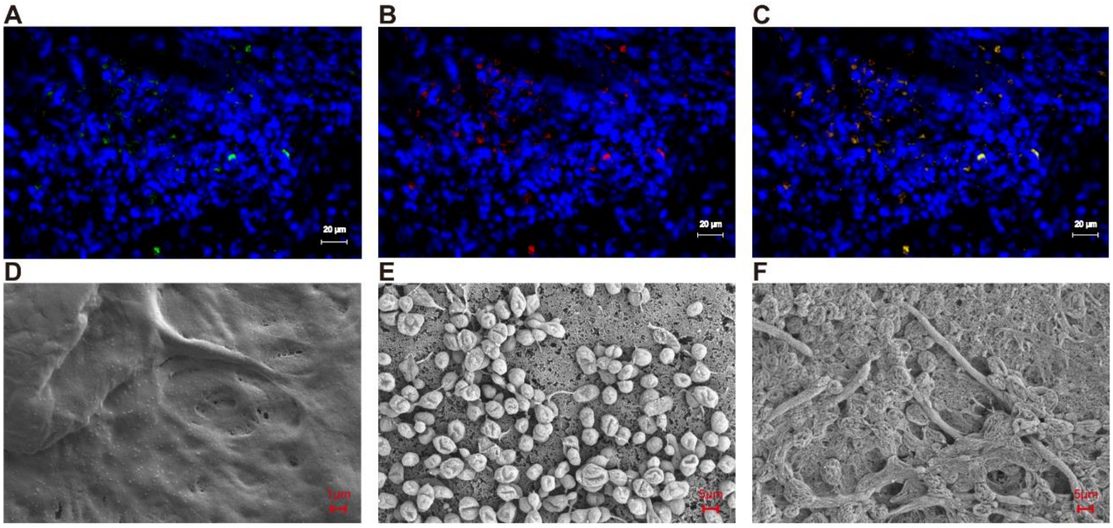

Supplement: Supplementary file 1 — Supplementary Material 1 [file 12885_2023_11439_MOESM1_ESM.pdf]
