# Supplementary material for: Fusobacterium nucleatum facilitates proliferation and autophagy by activating miR-361-3p/NUDT1 axis through oxidative stress in hypopharyngeal squamous cell carcinoma
Source: BMC Cancer. 2023 Oct 17;23:990. doi: 10.1186/s12885-023-11439-4 (PMC10580517; doi:10.1186/s12885-023-11439-4)

Fig S2. *Fn* reactivates the blockage of autophagy flow. The dashed line indicates that mCherry-LC3 puncta formation appeared after adding *Fn* in FaDu pre-treated with CQ.

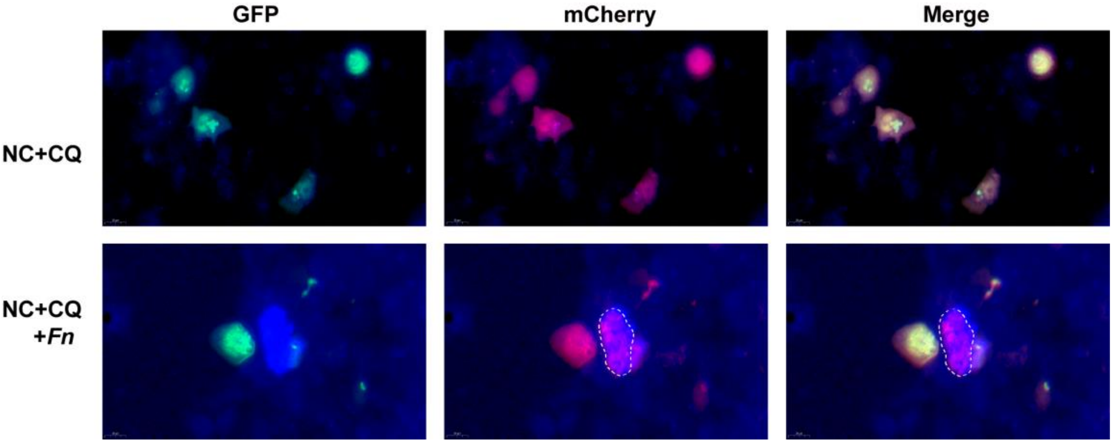

Supplement: Supplementary file 2 — Supplementary Material 2 [file 12885_2023_11439_MOESM2_ESM.pdf]
