# Supplementary material for: Fusobacterium nucleatum facilitates proliferation and autophagy by activating miR-361-3p/NUDT1 axis through oxidative stress in hypopharyngeal squamous cell carcinoma
Source: BMC Cancer. 2023 Oct 17;23:990. doi: 10.1186/s12885-023-11439-4 (PMC10580517; doi:10.1186/s12885-023-11439-4)

Fig S3. The distribution of NUDT1 in HPSCC patients and FaDu cells. (A-B) Nine HPSCC patients were enrolled. NUDT1 was verified higher in HC group. (C) EdU assay was used to supplement CCK-8 result.

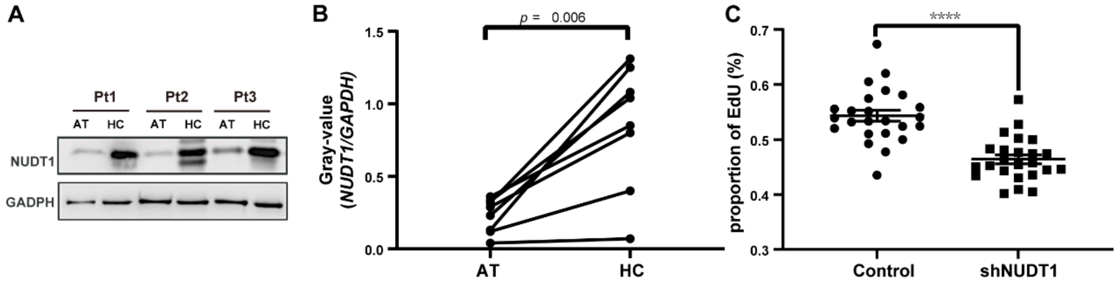

Supplement: Supplementary file 3 — Supplementary Material 3 [file 12885_2023_11439_MOESM3_ESM.pdf]
