# Supplementary material for: Fusobacterium nucleatum facilitates proliferation and autophagy by activating miR-361-3p/NUDT1 axis through oxidative stress in hypopharyngeal squamous cell carcinoma
Source: BMC Cancer. 2023 Oct 17;23:990. doi: 10.1186/s12885-023-11439-4 (PMC10580517; doi:10.1186/s12885-023-11439-4)

Fig S4. Differential effects of NUDT1 downregulation Induced by *Fn* on cell cycle and apoptosis. (A) The proportion of cells in the S-phase cell cycle in the *Fn*-treated group was observed to significantly increase relative to the control group. However, upon the downregulation of NUDT1, a notable reduction in the percentage of cells in the S phase was observed. (B) Despite the addition of *Fn* (MOI=100) to the FaDu cell population, no significant enhancement in apoptosis was recorded, but instead, shNUDT1 group saw upregulation. Thus, we hypothesize that DDR induced by *Fn* may be more attributed to autophagy.

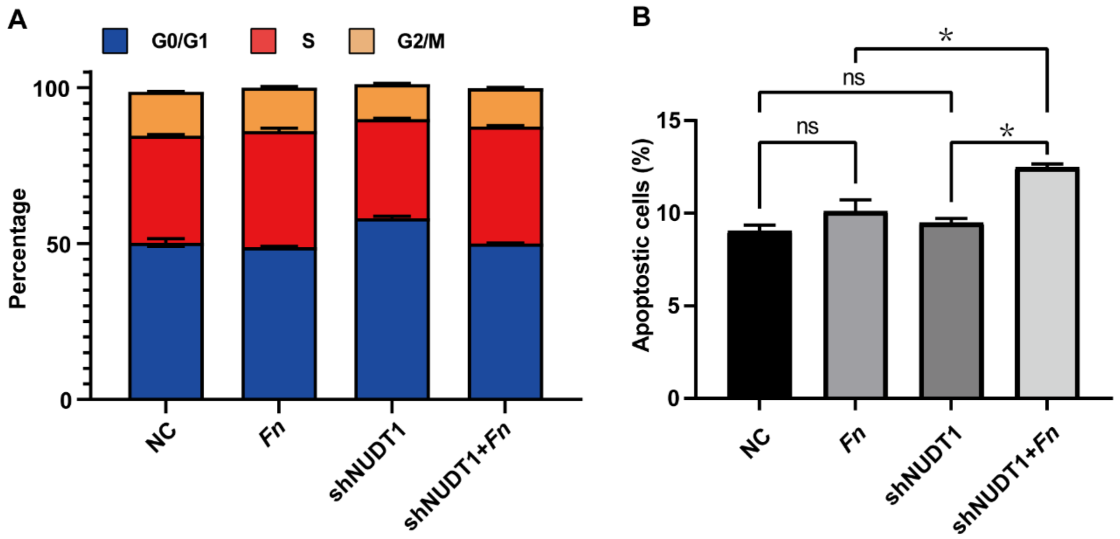

Supplement: Supplementary file 4 — Supplementary Material 4 [file 12885_2023_11439_MOESM4_ESM.pdf]
