# Supplementary material for: Fusobacterium nucleatum facilitates proliferation and autophagy by activating miR-361-3p/NUDT1 axis through oxidative stress in hypopharyngeal squamous cell carcinoma
Source: BMC Cancer. 2023 Oct 17;23:990. doi: 10.1186/s12885-023-11439-4 (PMC10580517; doi:10.1186/s12885-023-11439-4)
Supplement: Supplementary file 5 — Supplementary Material 5 [file 12885_2023_11439_MOESM5_ESM.pdf]

Table S1. Demographic of 82 enrolled HPSCC patients stratified with *Fn*

**Characteristics**	**Low *Fn* groups** N=58	**High *Fn* groups** N=24	***p*-value** (Two side)
Age (< 60 / ≥ 60)	27/31	12/12	0.776
Gender(female/male)	1/57	0/24	0.517
HTN (no/yes)	32/26	16/8	0.336
DM (no/yes)	52/6	22/2	0.780
Smoking (no/yes)	21/37	5/19	0.173
Alcohol (no/yes)	19/39	4/20	0.140
Pathological types (PS / nonPS)	49/9	22/2	0.385
IC before surgery (no/yes)	56/2	22/2	0.350
Options of operation (Pharyngectomy with/ without total Laryngectomy)	29/29	10/14	0.492
cT classification (T1-2/T3-4)	29/29	13/11	0.731
cN classification (N0/N+)	10/48	3/21	0.593
pT classification (T1-2/T3-4)	27/31	12/12	0.776
pN classification (N0/N+)	6/52	5/19	0.205
TNM stage (TNM Ⅲ/TNM Ⅳ)	11/47	7/17	0.310
ENE (- / +)	41/17	19/5	0.431
Tumor diameters (≤4cm/ >4cm)	45/13	17/7	0.517
Size of lymph node (≤3cm/ >3cm)	36/22	16/8	0.694

Two side *p*-value under Pearson or Exact chi-square test. $^*p < 0.05$ means statistical significance with bold marker. Abbreviation: DM, diabetes mellitus; HTN, hypertension; PS, pyriform sinus type; ENE, extranodal extension
