# Supplementary material for: Fusobacterium nucleatum facilitates proliferation and autophagy by activating miR-361-3p/NUDT1 axis through oxidative stress in hypopharyngeal squamous cell carcinoma
Source: BMC Cancer. 2023 Oct 17;23:990. doi: 10.1186/s12885-023-11439-4 (PMC10580517; doi:10.1186/s12885-023-11439-4)
Supplement: Supplementary file 6 — Supplementary Material 6 [file 12885_2023_11439_MOESM6_ESM.pdf]

Fig 3C Western-blot showed γH2AX increased after adding different MOI of *Fn*.

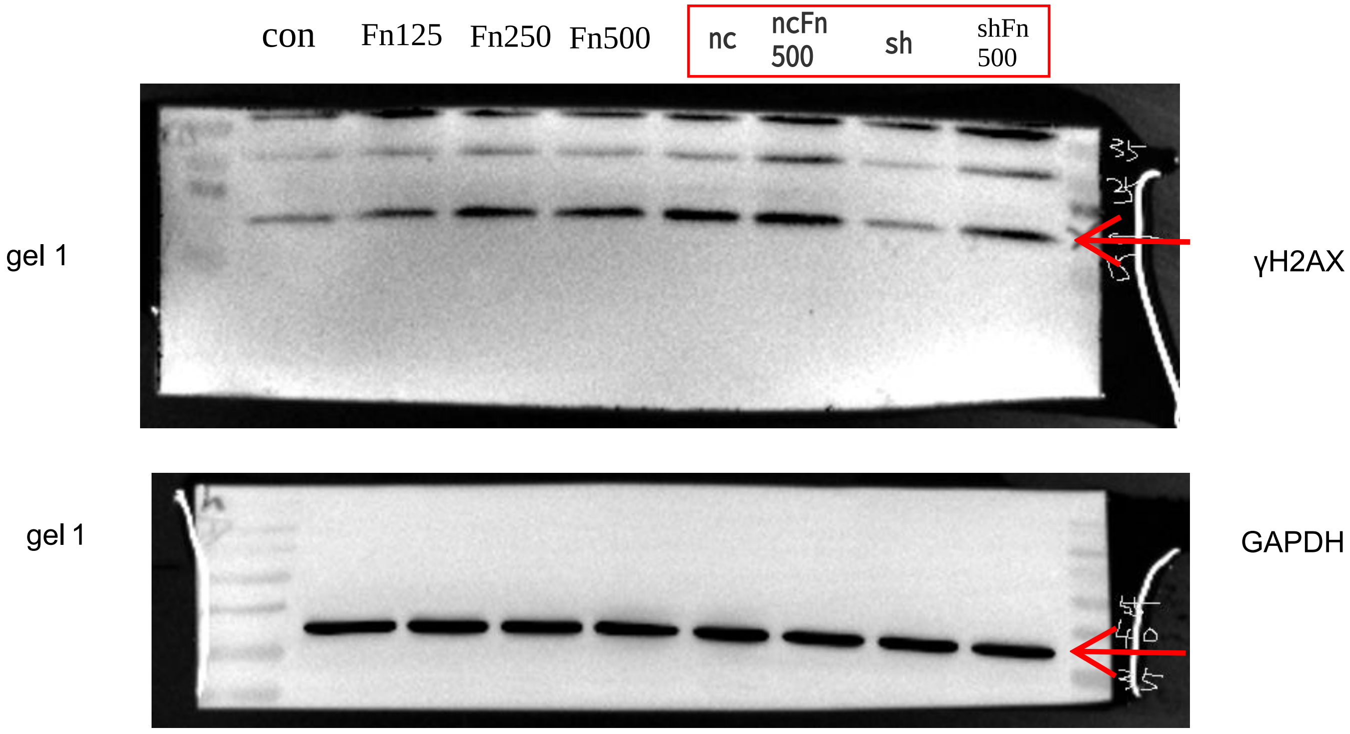

These four groups were cropped because we did not discuss the γH2AX expression when knocking down NUDT1(shNUDT1) here. sh means shNUDT1

Fig 5E Western-blot proved that PCNA was found downregulation in shNUDT1 as well.

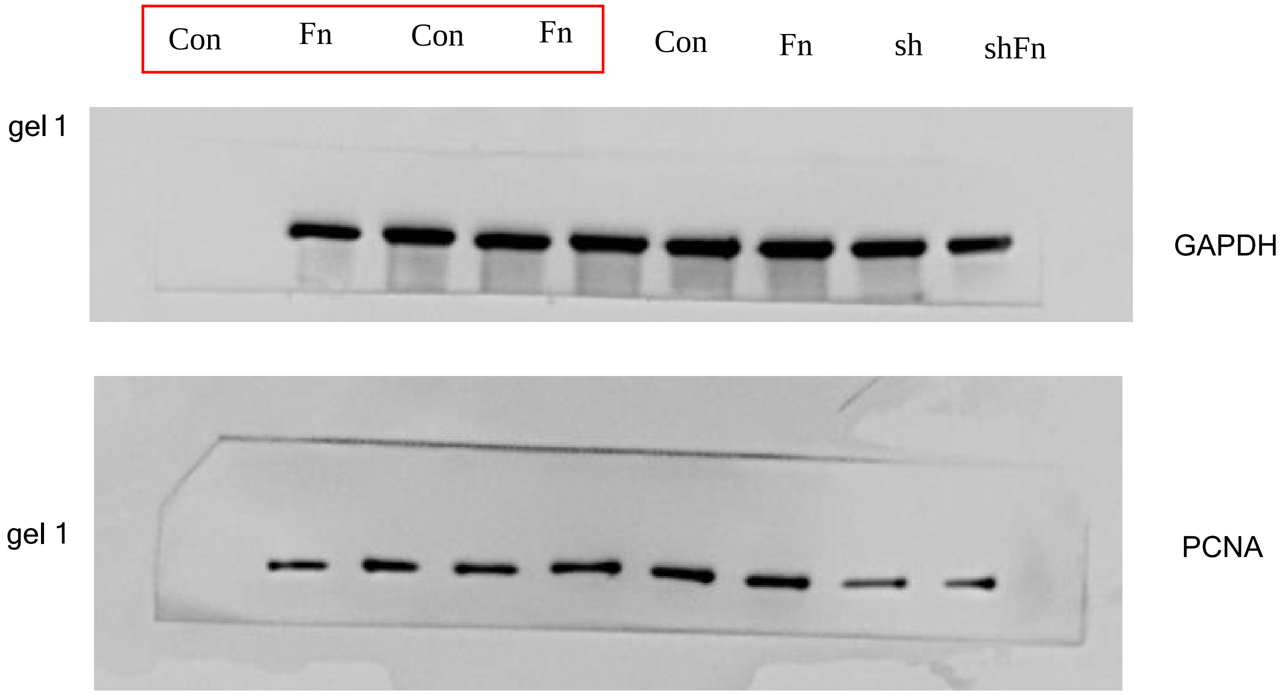

These replicates were cropped.

Fig 6C Western-blot proved that NUDT1 could be modulated by *Fn* triggered ROS.

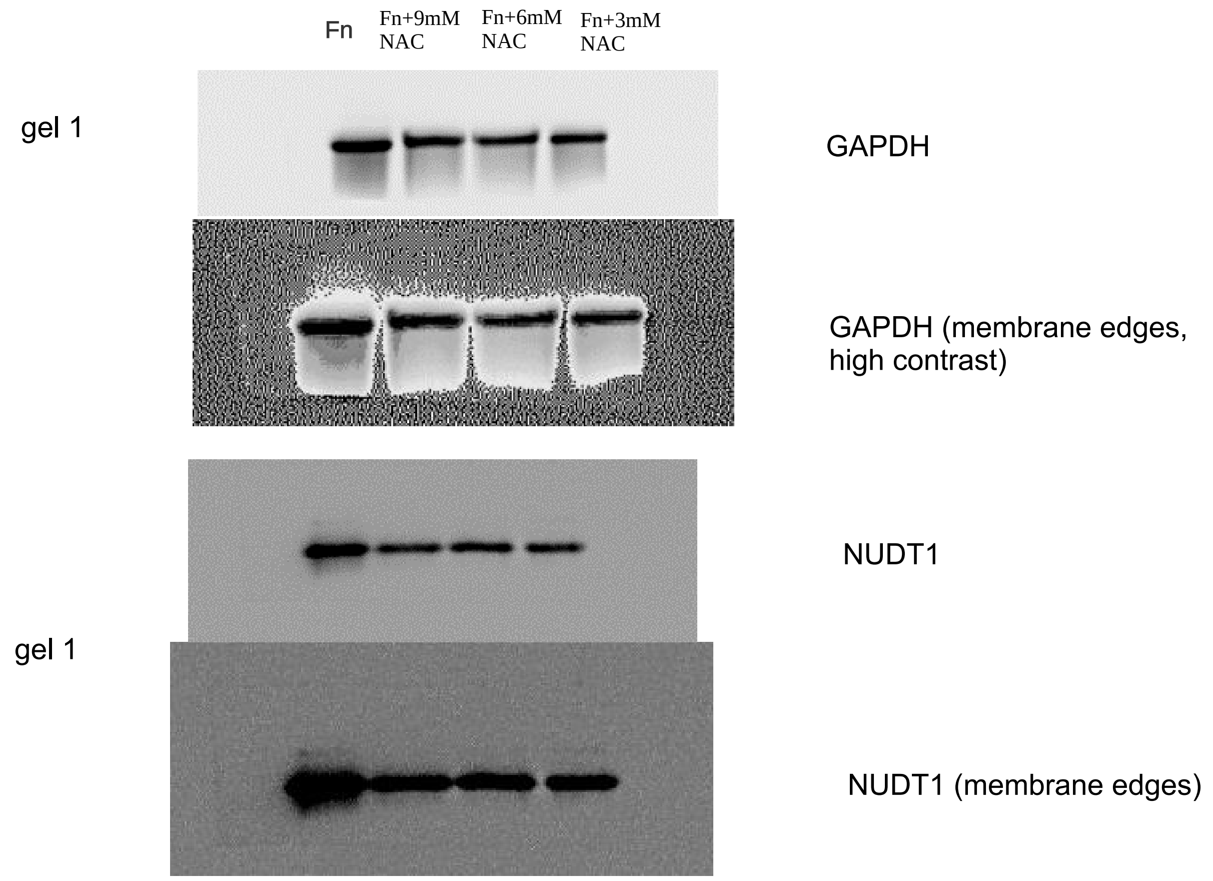

Fig 6D shNDUT1 group presents higher DDR, which showed that NUDT1 could repair DDR at some content.

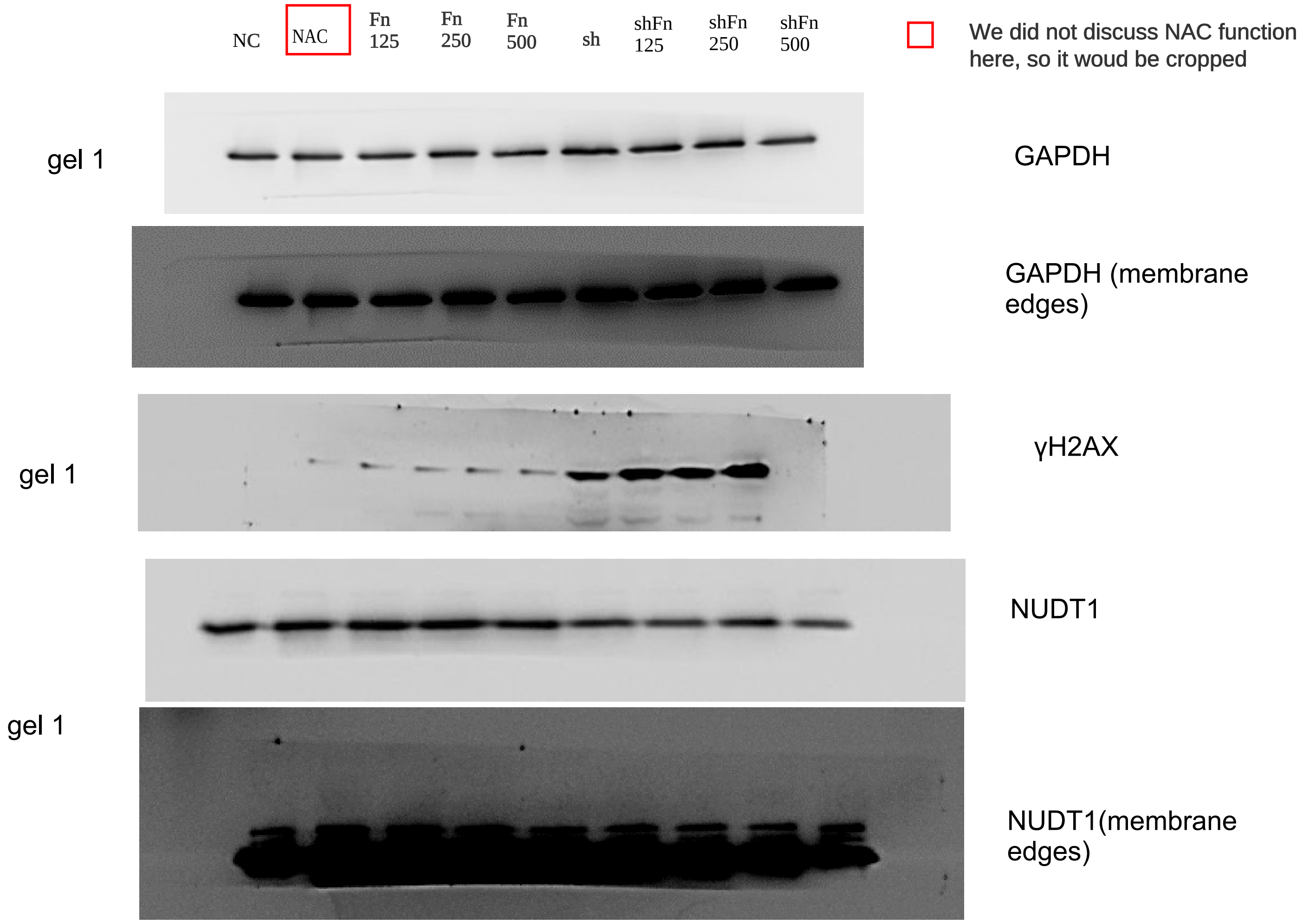

Fig 6E Autophagy-related protein was downregulated in shNUDT1. LC3B showed autophagy stagnant when shNUDT1 group co-cultured with *Fn* in the period of 2-12 hour.

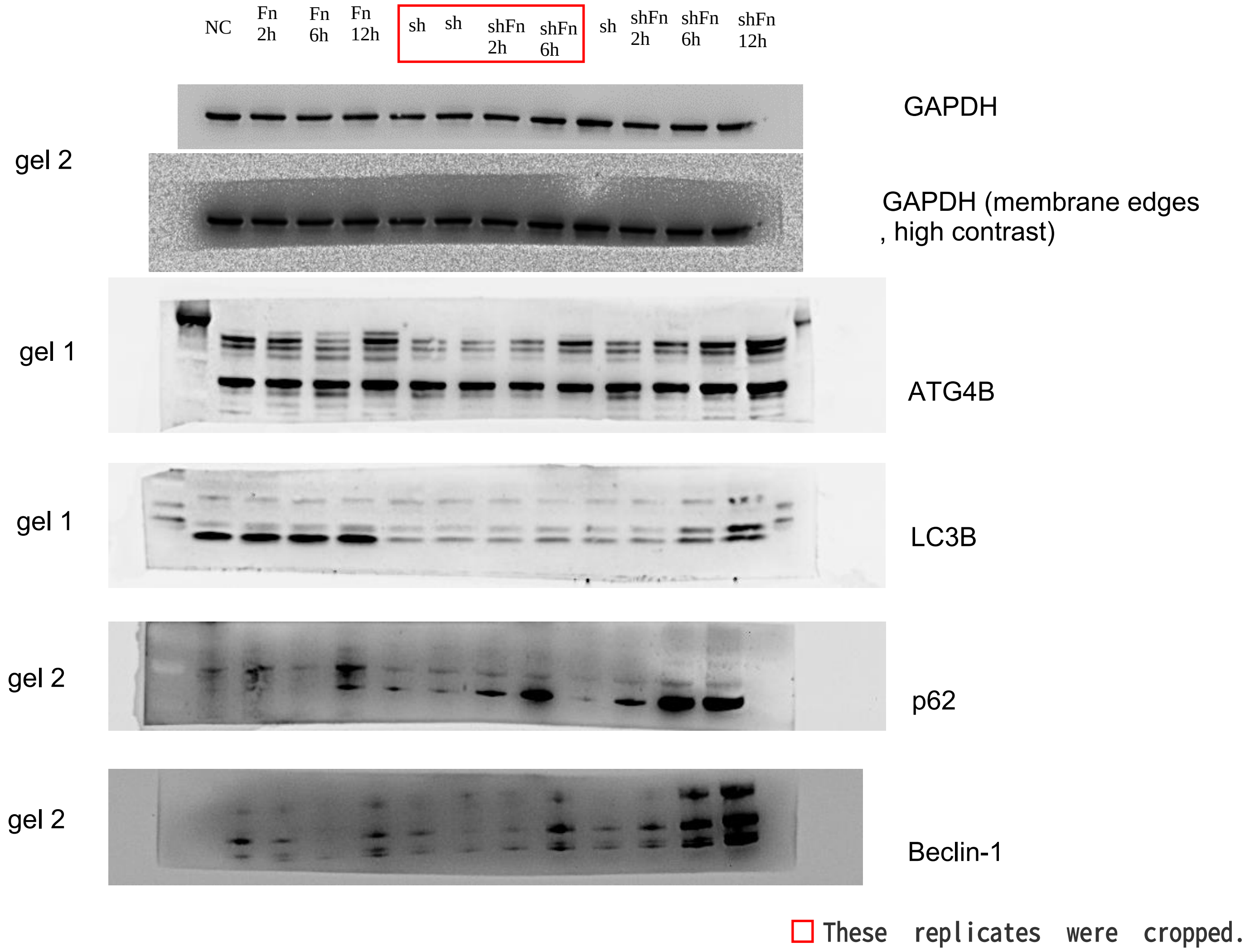

Fig 8A MiR-361-3p mimic could decrease the expression of NUDT1 and also LC3B.

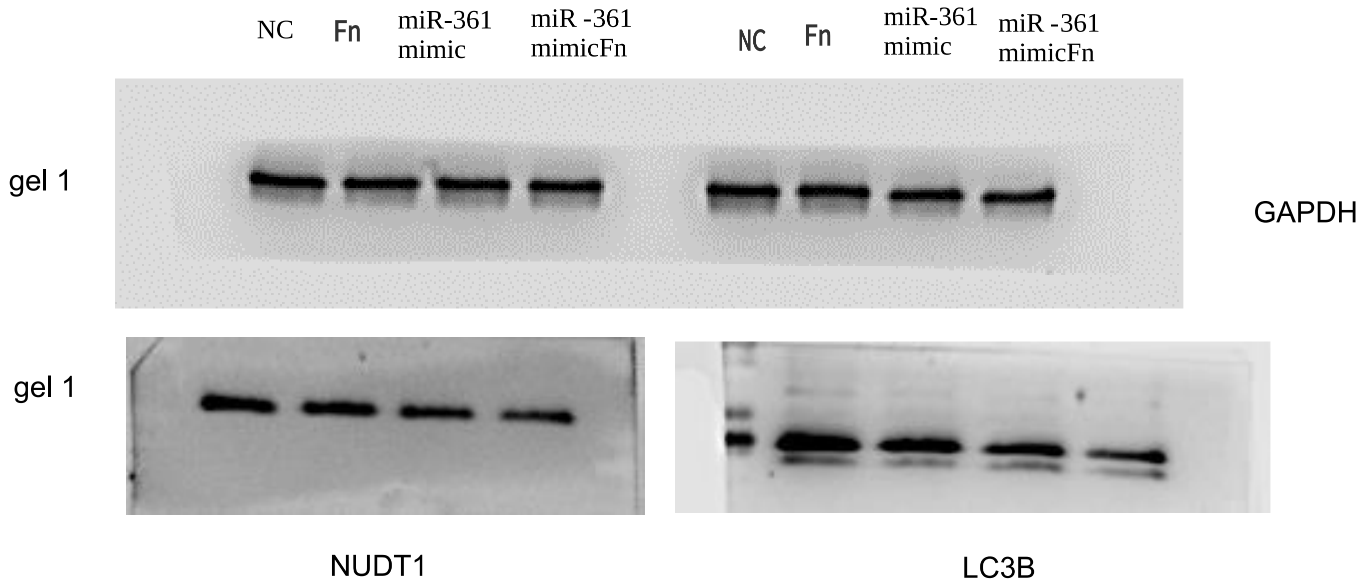

Fig S3A The distribution of NUDT1 in HPSCC patients (A- B) Nine HPSCC patients were enrolled.

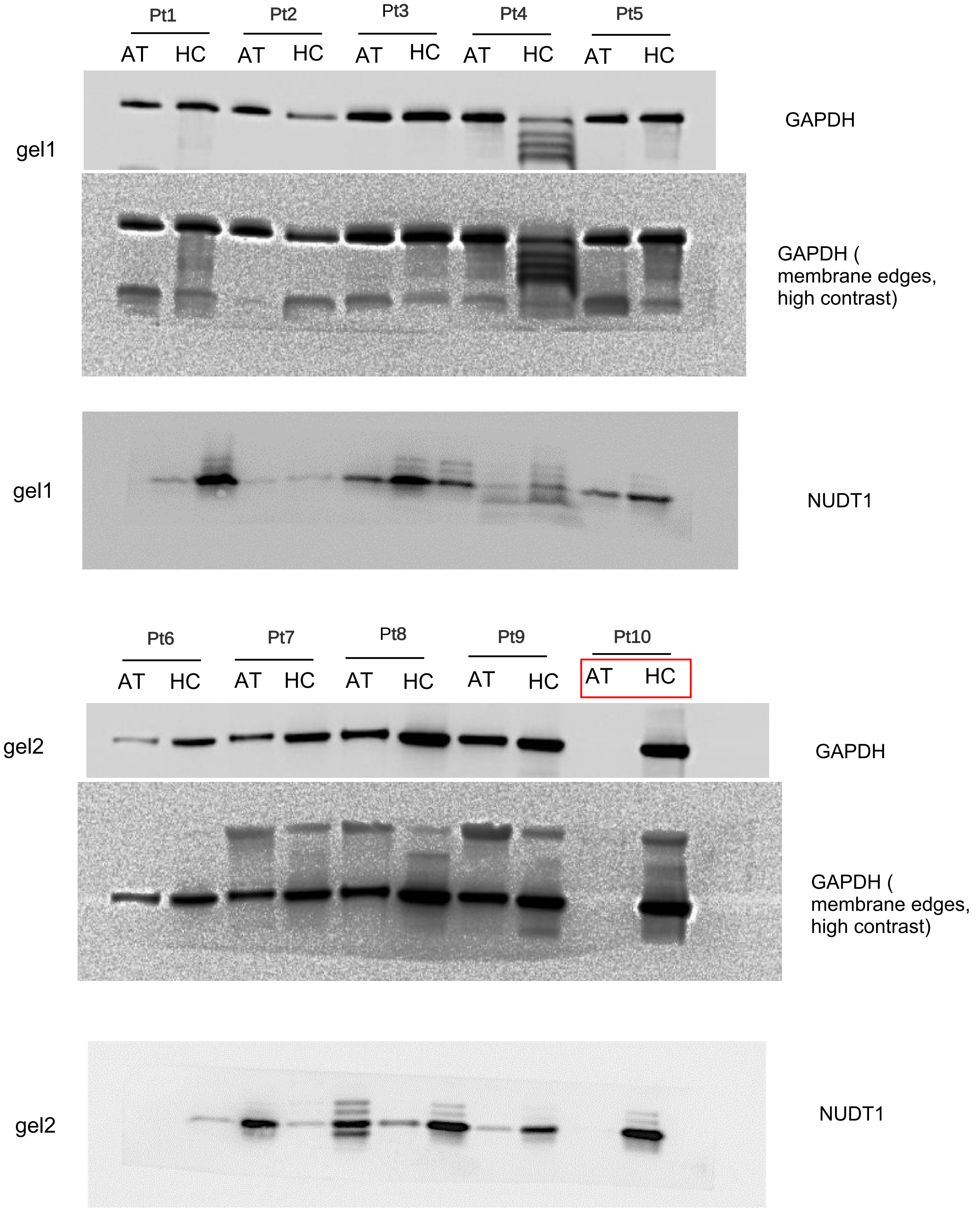

The GAPDH of one sample(Pt10) was absent,so this patient would be excluded.
